# Peer relationship, family factors, and mental health in rural children: a network analysis

**DOI:** 10.3389/fpsyt.2025.1708721

**Published:** 2025-12-15

**Authors:** Zhen-Xing Huang, Hao-Ming Li

**Affiliations:** Wenzhou Seventh People’s Hospital, Wenzhou, China

**Keywords:** anxiety, depression, network analysis, peer relationship, rural adolescent

## Abstract

The transition from childhood to early adolescence is a critical developmental period, particularly in rural areas where unique factors play a significant role. This study examined the interplay of peer and family factors on the mental health, including anxiety and depression, of 694 rural Chinese children in the transition to adolescence (aged 10-14). This study employed a quantitative design, analyzing data from 16 rural schools across seven provinces using Mixed Graphical Model (MGM) and Gaussian Graphical Model (GGM) network analyses to explore the relationships between variables related to family factors, peer relationships, anxiety, depression, and behavioral problems. Network analysis revealed that depression was a central node, and peer relationships had a greater impact on the mental health of these children than family factors. There was a significant correlation between depression and anxiety (weight = 0.55). Peer relationships were negatively associated with depression (weight = -0.31) and positively associated with father involvement. In contrast, the network shows that the experience of being left behind was not directly associated with mental health outcomes. Further analysis of the depression network revealed that negative mood was a central node. This study highlights that peer relationships have a far stronger connection to the mental health of rural Chinese children than family factors. Depression, and specifically negative mood, was central to the network, emphasizing the need for interventions that focus on improving peer interactions and addressing core depressive symptoms.

## 1 Introduction

The transition from childhood to early adolescence is a critical developmental period marked by significant emotional, social, and psychological changes. This stage is foundational, often establishing mental health patterns that persist into adulthood (1). For children in rural China, this transition is embedded within a context of distinctive socio-cultural and economic factors, creating developmental trajectories that differ markedly from those of their urban peers. A growing body of evidence indicates that rural children may experience higher rates of mental health challenges, including depression and anxiety, compared to their urban counterparts (2). These disparities are frequently linked to unique contextual factors, such as limited access to mental health resources, distinct cultural norms, and specific family dynamics shaped by widespread parental migration (3).

Within this context, family dynamics have long been recognized as a pivotal determinant of psychological well-being (4). Positive parental relationships, characterized by warmth, effective communication, and consistent involvement, are robustly associated with reduced risks of internalizing disorders in children. However, the common phenomenon of parental migration for work in rural China introduces significant challenges to these family dynamics (5). While much literature has focused on the “left-behind” experience, linking it to higher levels of depression and anxiety (6), it is crucial to understand this as one factor within a broader familial system. For instance, the role of father involvement has emerged as a critical factor, with studies indicating that positive paternal engagement can significantly buffer against familial stressors and promote social adjustment, even in the context of migration (7).

Concurrently with these family influences, early adolescence is characterized by a profound developmental shift toward peer-centric social worlds (8). Peer relationships become a primary context for social and emotional development, acting as key conduits for social support, identity formation, and behavioral norms (9). The quality of these interactions has a direct and significant association with mental health; positive peer acceptance and support are linked to better psychological outcomes, whereas peer rejection and victimization can exacerbate distress (10). In the specific context of rural China, these peer networks may play an especially crucial buffering role. Research suggests that supportive peer relationships can mitigate the negative impacts of family adversity, such as the distress associated with parental absence (11).

Despite the well-documented importance of both family and peer domains, a significant gap persists in the literature. The majority of existing studies tend to examine either family or peer influences in isolation, presupposing one or the other as the primary driver of mental health outcomes. This siloed approach fails to capture the complex interplay and, critically, the relative importance of these two social worlds as they co-exist in a child’s daily life. It is particularly unclear how these systems interact for rural children, who must simultaneously navigate factors like parental absence while relying heavily on their immediate, and often highly stable, school-based peer groups. This study seeks to address this gap by conceptualizing these influences not as independent predictors, but as an interconnected system.

To explore this complex system, we employ a network analysis approach. Unlike traditional regression models that focus on linear, unidirectional associations, network analysis provides a holistic, data-driven visualization of the entire web of reciprocal relationships (12). This method allows us to quantify the intricate connections among multiple psychosocial variables simultaneously, identifying which factors are most central and how they directly and indirectly influence one another. Network analysis has emerged as a powerful tool in adolescent mental health research. For instance, Sánchez Hernández et al. (2023) (13) applied network analysis to explore the comorbidity of anxiety and depression symptoms in Spanish children, identifying ‘worry’ and ‘fatigue’ as key bridge symptoms connecting the two disorders. Similarly, Long et al. (2020) (14) employed this approach to investigate associations between mental health and peer relationships, demonstrating that while depression was linked to perceived social isolation, conduct disorders were more related to having antisocial peers. These studies demonstrate the utility of network analysis in understanding complex psychosocial dynamics.

The current study, therefore, utilizes network analysis to examine the intricate system of peer relationships, family factors (including parental relationships and father involvement), and mental health outcomes (depression, anxiety, and behavioral problems) in a large sample of rural Chinese children. Grounded in social development theory, which posits the increasing salience of peers during adolescence (15), we hypothesized that peer relationships would emerge as a highly central component of this psychological network, potentially exerting a stronger influence than family factors. Furthermore, we expected to observe strong comorbidity between depression and anxiety. We also anticipated that the left-behind experience, while an important contextual variable, might not show a direct connection to mental health outcomes, but rather be mediated by more proximal factors within the network, such as the quality of peer and family relationships.

## 2 Method

### 2.1 Data source

This study employed a quantitative cross-sectional design, analyzing secondary data from the ‘Elementary School Students’ Living Conditions Survey’ conducted in 2021 (16). A multi-stage sampling strategy was utilized by the original researchers to ensure a representative sample of rural Chinese students. Initially, 16 rural schools were selected from seven provinces across diverse geographical and socioeconomic regions of China: Anhui, Gansu, Guangdong, Heilongjiang, Hubei, Hunan, and Sichuan.

All students from grades one through six at these schools were invited to participate. From 3,025 distributed questionnaires, 2,498 valid responses were obtained (an 82.28% effective response rate) after excluding those with significant missing data or careless responding. For the purpose of the present analysis, the sample was necessarily restricted to participants in grades five and six. This selection criterion was dictated by the original data collection protocol, in which key psychological instruments were administered only to the older students (grades 5-6) due to the age appropriateness of the scale and to reduce response burden. Our final analysis sample thus comprises 694 participants (aged 10–14 years; Mean [M] = 12.15, Standard Deviation [SD] = 0.84) for whom complete data were available on all core variables included in our network model. This age range represents a critical developmental period spanning late childhood and the onset of early adolescence.

### 2.2 Procedure and ethical considerations

Data collection was conducted from March to May 2021 under the supervision of professionally trained personnel. The survey, which utilized standardized questionnaires to assess students’ psychological well-being, family dynamics, and peer relationships, was administered in classroom settings. Prior to administration, supervisors thoroughly explained the study’s purpose and procedures to participants. They remained present throughout the survey administration to maintain classroom order, answer queries, and ensure smooth implementation.

The study adhered to strict ethical guidelines aligned with the Helsinki Declaration of 1975 (as revised in 2000). Although direct ethical approval for the current analysis was not required due to the use of existing, de-identified data, the original data collection received approval from the institutional ethics committee (approval number: CAS-WX2021PY-0204). Informed consent was obtained from all participants before their involvement. During data processing, all personal information was anonymized to protect participant privacy. The data preparation phase included recoding reverse-scored items, calculating scores for each research variable, and generating new variables for analysis. A comprehensive review of the entire dataset was conducted to ensure accuracy and data integrity.

### 2.3 Measures

#### 2.3.1 Demographic information

Participants provided information on their sex, age, academic grade level (Gr), only child status (OCS), and left-behind child status (LB). They also reported on the perceived quality of parental relationships (PR), rated on a 5-point scale from 1 (Very Harmonious) to 5 (Very Disharmonious); desired educational attainment (DEA); and preference for future work location (PFWL).

#### 2.3.2 Generalized anxiety disorder scale

The 7-item Generalized Anxiety Disorder (GAD-7) scale (17) was used to assess anxiety symptoms. The Chinese version uses a 4-point scoring system (0–3), with total scores ranging from 0 to 21 (18). The scale demonstrated good internal consistency in this study (α = .81).

#### 2.3.3 Child behavior checklist

Child problem behaviors were assessed using a 20-item scale derived from the Rule-Breaking Behavior subscale of the Achenbach CBCL (19). Items are rated on a 3-point scale (0–2), with higher scores indicating greater problem behaviors. The Chinese version showed good reliability (α = .80) (20).

#### 2.3.4 Father Involvement questionnaire

The FIQ is a 22-item scale evaluating paternal involvement in areas such as care, academic support, and emotional communication (21). Items are scored from 0 to 4, with higher total scores indicating greater involvement. The scale exhibited excellent reliability in this study (α = .94).

#### 2.3.5 Peer relationship scale

The PRS is an 8-item tool assessing the quality of peer relationships (22). Items are rated on a 5-point scale (scores range 0–32), with higher scores signifying better relationships. It demonstrated good reliability (α = .86) in this study.

#### 2.3.6 Child depression inventory

The CDI is a 27-item scale that evaluates the emotional, cognitive, and behavioral aspects of depression in children (23). Items are scored on a 3-point scale (0–2), with total scores ranging from 0 to 54. The Chinese version of the Children’s Depression Inventory (CDI-C) showed good internal consistency in this survey (α = .88) (24).

### 2.4 Statistics analysis

Mixed Graphical Models (MGMs) are designed to analyze datasets that include both continuous and discrete variables. They offer a flexible approach suitable for high-dimensional data. In MGMs, a computationally efficient regression-based algorithm is used for model fitting, focusing on the conditional log-likelihood of each variable given the rest. The parameters of MGMs have a natural group structure, and sparsity in the fitted graph is achieved by incorporating a group lasso penalty, which is approximated by a weighted lasso penalty for computational efficiency. Further, we employed a Gaussian Graphical Model (GGM) to delve into CDI structure. We chose a λ-regularization parameter based on the Extended Bayesian Information Criterion (EBIC) for graph fitting. Key centrality measures—strength, closeness, betweenness, and expected influence—were calculated to identify influential nodes. Stability and robustness of our findings were ensured through a case-dropping bootstrap approach. In our study, we used ‘mgm’, ‘bootnet’, and ‘estimateNetworkpackages’ in R.

## 3 Result

### 3.1 Descriptive statistics

**Table 1** presents descriptive statistics for the key measurements in our study, stratified by demographic variables. For instance, female participants showed slightly higher mean scores on both GAD and CDI compared to male participants. Left-behind children exhibited marginally higher scores on both GAD and CDI compared to non-left-behind children. Notably, parental relationship quality showed a clear trend, with children reporting very harmonious parental relationships having the lowest mean scores on both GAD and CDI, while those reporting not very harmonious relationships had the highest mean scores.

**Table 1:** Descriptive statistics for the measurements on rural children.

Democratic variables	Levels	*N*	GAD		CDI	
			*M*	*SD*	*M*	*SD*
Sex	Female	380	6.51	4.79	15.37	8.33
	Male	327	5.76	4.75	13.70	7.73
Only_Child_Status	No	618	6.13	4.72	14.46	8.09
	Yes	89	6.39	5.21	15.61	8.06
Left_Behind	No	516	6.03	4.78	14.30	7.89
	Yes	191	6.52	4.78	15.41	8.58
Grade	Five	334	6.20	4.76	14.16	7.75
	Six	373	6.13	4.81	14.99	8.38
Parental_Relationship	Very Harmonious	315	4.81	4.31	11.49	6.91
	Relatively Harmonious	202	6.45	4.51	15.14	7.40
	Average	116	7.97	4.66	18.59	7.49
	Not Very Harmonious	46	8.93	5.93	20.63	9.18
	Very Disharmonious	28	7.36	5.41	19.36	10.16

### 3.2 Network analysis using MGMs

**Figure 1:**
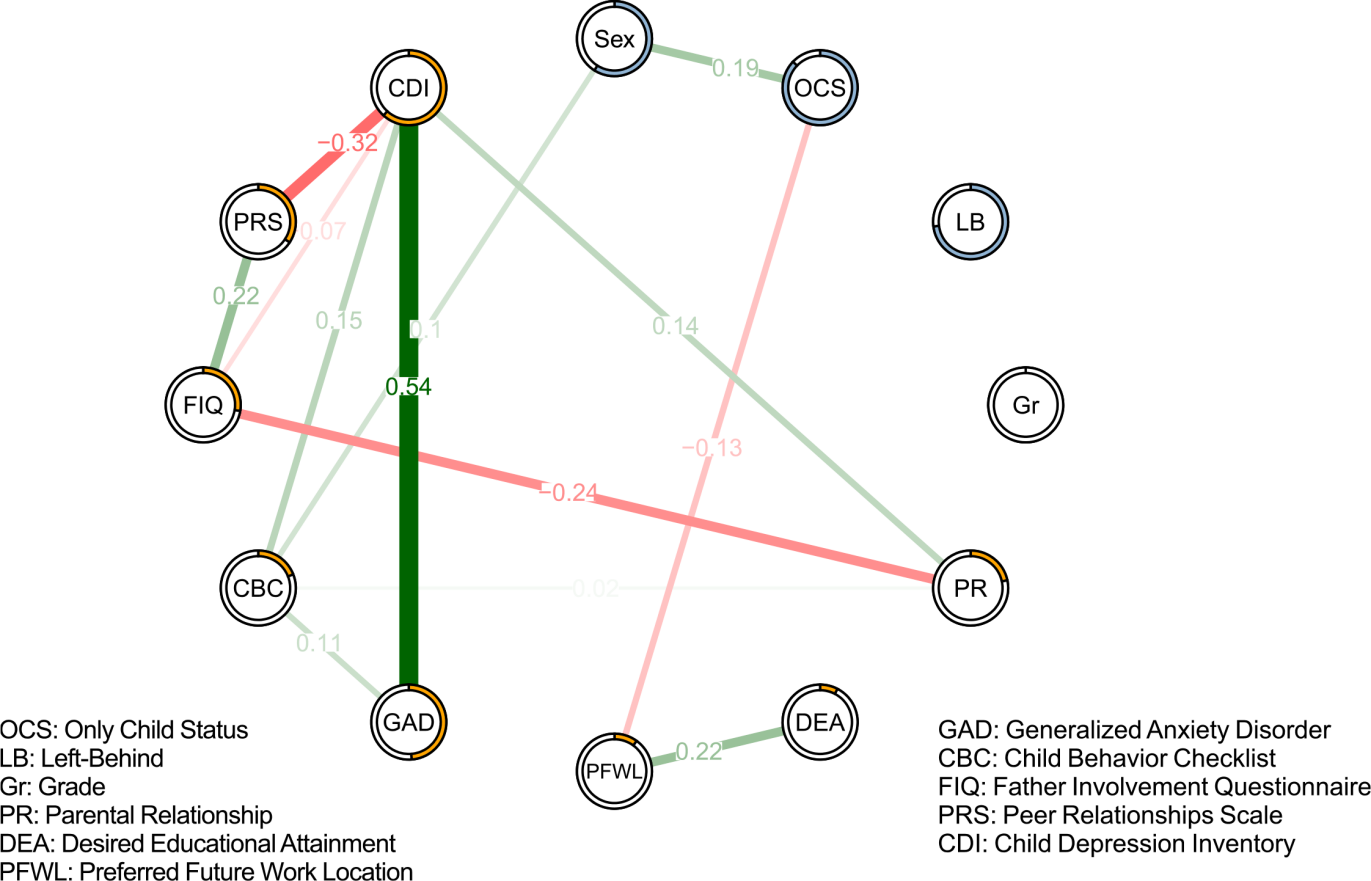
Estimated network structure of 694 children based on the *mgm* package in R. Green/red edges indicate positive/negative weights between nodes. Rings on nodes indicate R^2^ (continuous variable, orange) or categorical accuracy (categorical variable, light blue).

A Mixed Graphical Model (MGM) was estimated to explore the relationships between family factors (FIQ, PR), peer relationships (PRS), mental health outcomes (CDI, GAD, CBCL), and demographic variables. The resulting network structure was sparse, with only 13 of 66 possible edges showing non-zero weights after regularization. The network structure is visualized in **Figure 1**. Several key connections emerged. A strong positive association was found between the Child Depression Inventory (CDI) and the Generalized Anxiety Disorder (GAD) scale, with a weight of 0.55. The Peer Relationship Scale (PRS) was negatively associated with CDI (weight = -0.31) and positively associated with the Father Involvement Questionnaire (FIQ) (weight = 0.22). In turn, FIQ was negatively associated with Parental Relationship quality (PR) (weight = -0.22); it is important to note that the PR scale was reverse-coded, with higher scores indicating less harmonious relationships. Notably, the ‘Left-Behind’ (LB) status variable showed no direct connections to any mental health outcomes in the final network.

Despite the significant mean differences found in the descriptive t-tests, gender did not emerge as a significant node in the network analysis after the weighted lasso penalty was applied. This suggests that while mean-level differences exist, gender was not a robust structural connector to other variables once the full system of interactions was modeled.

**Figure 2:**
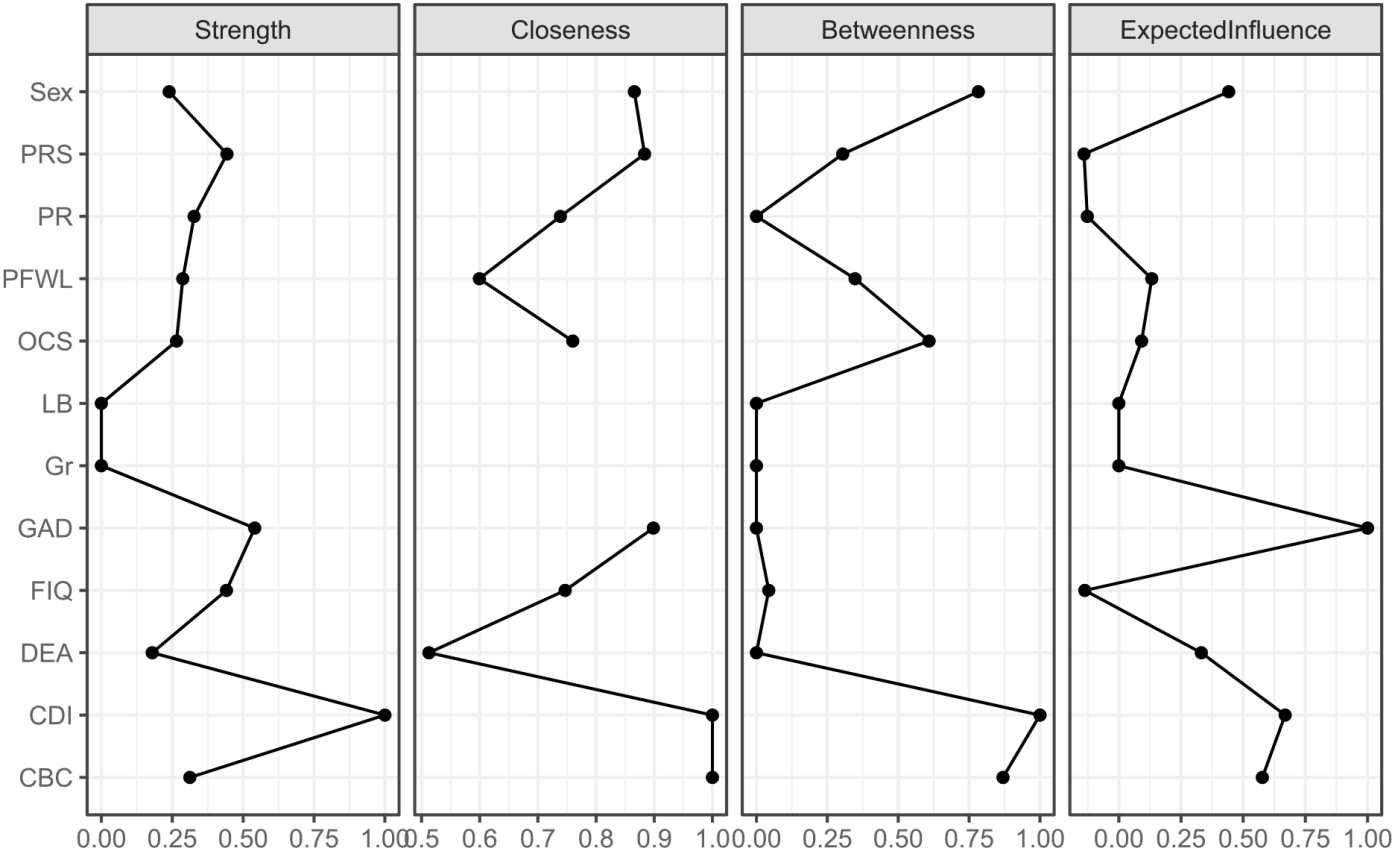
Centrality indices for the nodes of the present network, including those for strength, betweenness, closeness, and expected influence. The full names of the abbreviations can be found in **Figure 1**.

**Figure 3:**
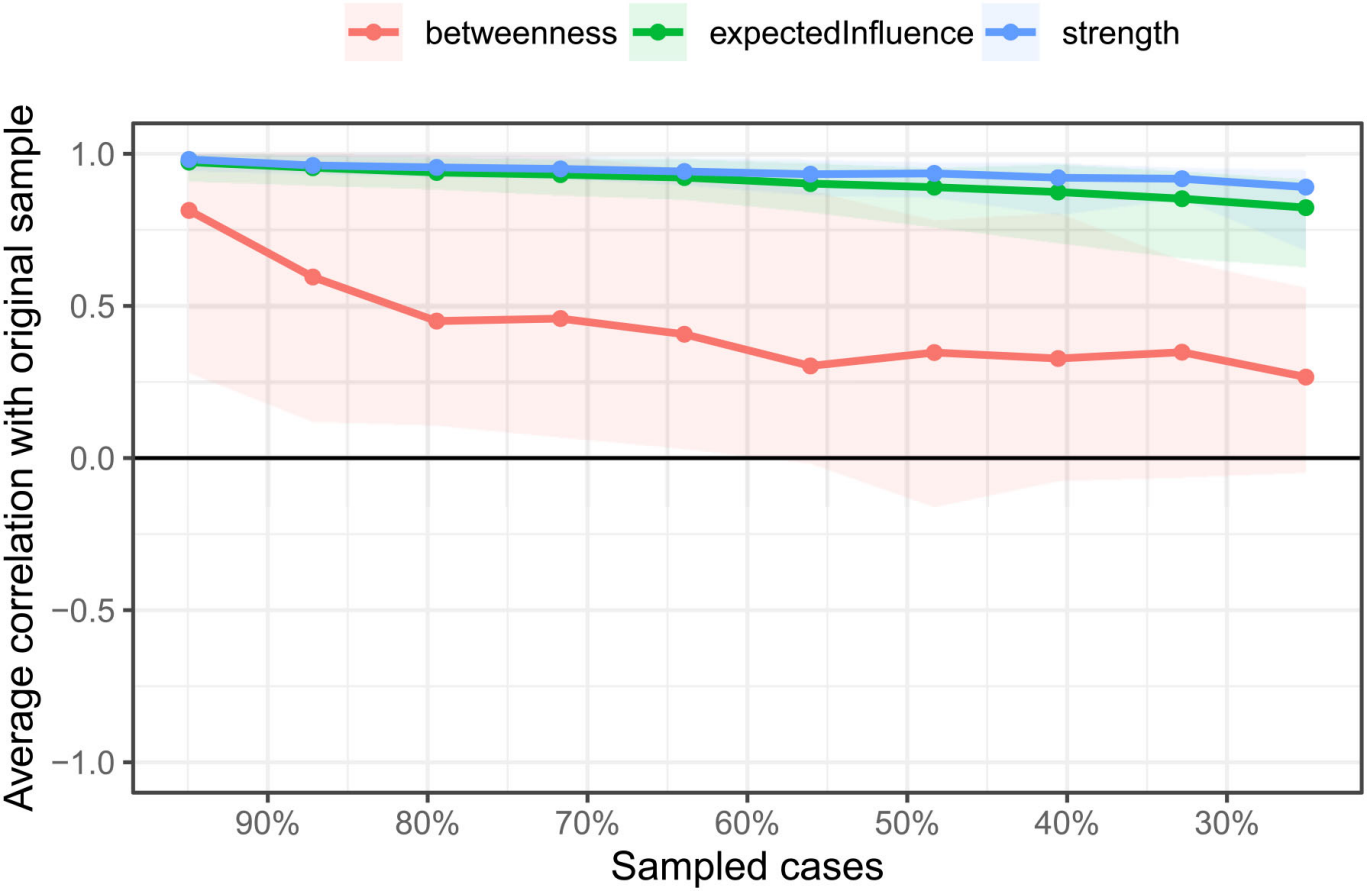
The average correlation coefficients between selected cases and the full sample for the centrality indices of networks. Lines indicate the means and areas ranging from the 2.5th quantile to the 97.5th quantile.

Centrality indices—strength, closeness, betweenness, and expected influence—were calculated to identify the most influential nodes in the network (see **Figure 2**). The CDI node exhibited the highest centrality in terms of strength, closeness, and betweenness, indicating its critical role in network connectivity. GAD also demonstrated substantial influence, followed by PRS. CDI did not, however, have the highest expected influence, which accounts for the direction and weight of connections. The stability of the network was assessed using a case-dropping bootstrap approach (see **Figure 3**). The Correlation Stability (CS) coefficient indicated high stability for strength (CS[cor = 0.7] = 0.75) and expected influence (CS[cor = 0.7] = 0.62), both of which are above the recommended 0.5 threshold. The betweenness index, however, was found to be unstable (CS[cor = 0.7] = 0), likely due to the sparse nature of the network and multiple disconnections between variables.

### 3.3 Depression symptom network analysis

**Figure 4:**
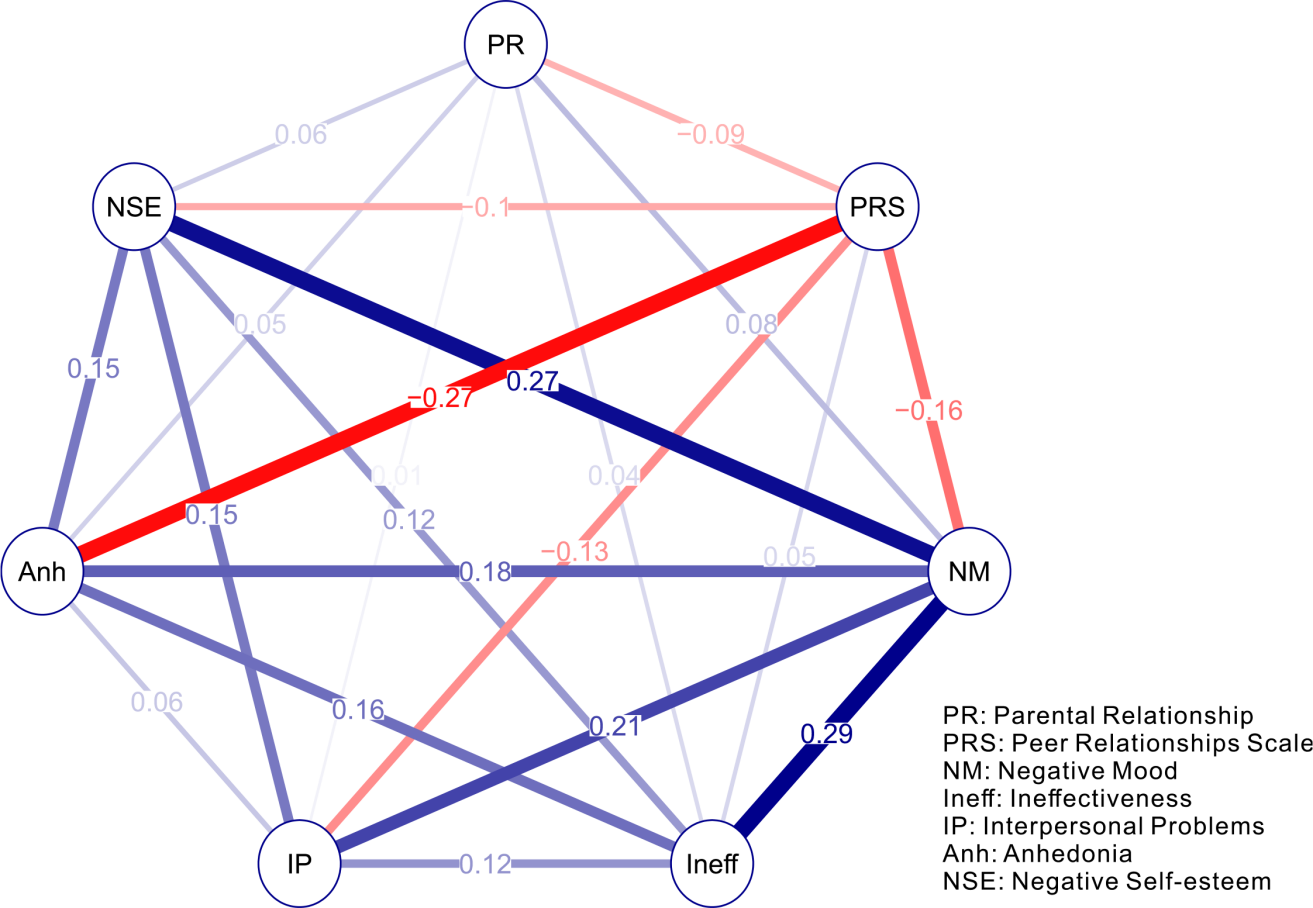
Estimated network structure of 694 children based on the *estimateNetwork* package in R. Blue/red edges indicate positive/negative weights between nodes.

Given the high centrality of the CDI node, a secondary Gaussian Graphical Model (GGM) was estimated to explore the internal structure of depression symptoms, along with their specific connections to Peer Relationships (PRS) and Parental Relationships (PR). This network is presented in **Figure 4**. The resulting network was fully connected, with all 21 edges bearing significant weights, indicating no regularization was necessary. A notable finding within this symptom-level network was a strong negative relationship between peer relationships (PRS) and anhedonia (weight = -0.27).

**Figure 5:**
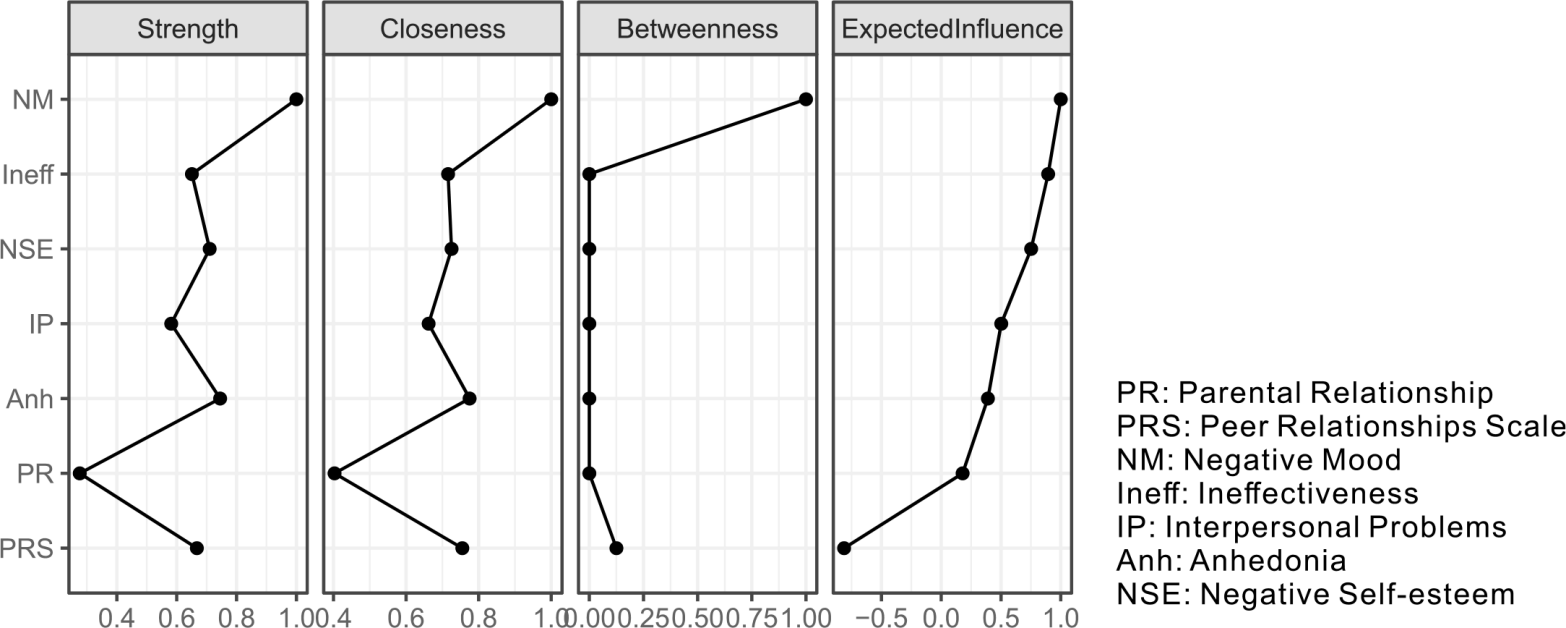
Centrality indices for the nodes of the present network, including those for strength, betweenness, closeness, and expected influence.

Centrality analysis of the depression symptom network (**Figure 5**) revealed that Negative Mood (NM) was the most central node, exhibiting the highest scores across all calculated metrics (strength, closeness, betweenness, and eigenvector centrality). This underscores its pivotal role within the depression symptom structure. Furthermore, when comparing the two social variables, the peer relationship (PRS) node was more central than the parental relationship (PR) node according to strength, closeness, and betweenness metrics.

**Figure 6:**
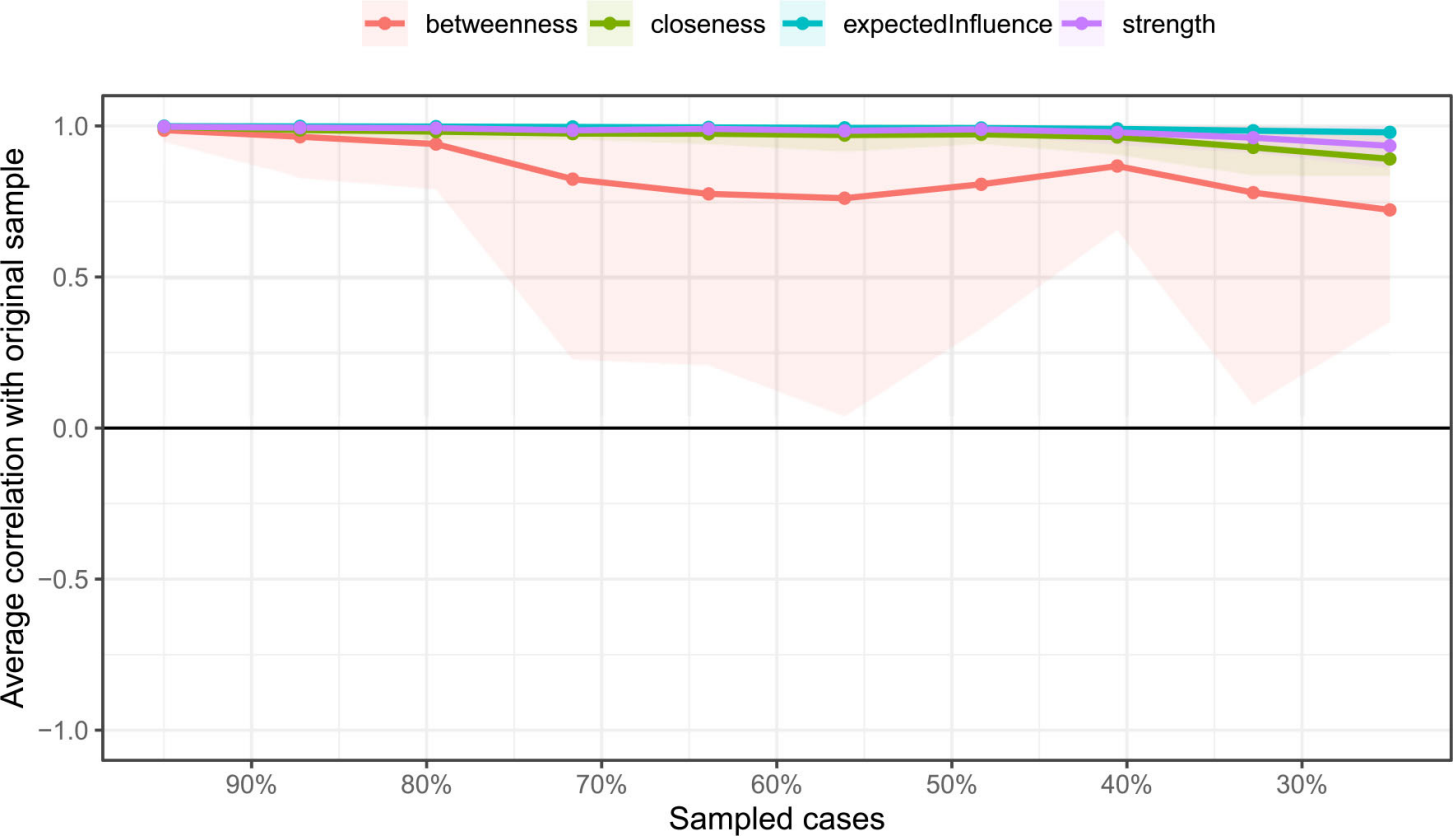
The average correlation coefficients between selected cases and the full sample for the centrality indices of networks. Lines indicate the means and areas ranging from the 2.5th quantile to the 97.5th quantile.

The stability of this second network (**Figure 6**) was found to be highly robust. The CS coefficients were high for betweenness (CS[cor = 0.7] = 0.672) and exceptionally high for both strength and eigenvector centrality (CS[cor = 0.7] = 0.75), indicating that the network attributes are reliable.

## 4 Discussion

Our network analysis of 694 rural Chinese children offers a nuanced perspective on the architecture of mental health during early adolescence, highlighting the central role of peer relationships. Grounded in social development theory, our central finding is that peer relationships exerted a stronger relationship to psychological well-being than family factors. This aligns with an ecological systems perspective (25), suggesting that in the specific context of rural China—where parental migration is common—the peer microsystem may become a primary source of social and emotional support. In this integrated discussion, we explore how this central finding contextualizes our other key results, including the strong comorbidity between depression and anxiety, the centrality of negative mood, and the notable absence of a direct link between left-behind status and mental health outcomes.

The preeminence of peer relationships over family factors in our network is a critical finding. This may be amplified by the unique social structures of rural Chinese schools, which often feature more stable and cohesive populations compared to more transient urban environments (26). These long-term, stable peer cohorts can foster deep connections that serve as a primary and reliable source of emotional support, becoming particularly salient when traditional family support is less consistent due to parental migration. Furthermore, this finding may reflect an ongoing sociocultural transformation in China. While traditional Chinese culture emphasizes familial hierarchy (27), rapid modernization may be elevating the role of peer groups as a powerful force for socialization and identity formation, even in rural settings.

One of the most intriguing findings of our study is the minimal direct impact of the left-behind experience on mental health outcomes. While extensive literature documents the adverse psychological effects of parental migration (28, 29), our results suggest a more complex reality. Rather than attributing this to an unmeasured construct like resilience, our network analysis points to a potential explanation within the data itself: the powerful, buffering influence of peer relationships. It is highly plausible that in these rural communities, supportive and stable peer networks serve as a critical protective factor, mitigating the potential loneliness or distress associated with parental absence. Our finding that peer relationships are highly central to well-being supports this interpretation. This suggests the relation to being “left-behind” is not absent, but is likely indirect and mediated by the quality of these more proximal peer and remaining familial connections, a hypothesis that future longitudinal and mediation analyses should explore.

Beyond the social context, our findings illuminate the internal structure of psychopathology in this population. The network revealed a robust positive edge between depression and anxiety, corroborating the high comorbidity consistently found in adolescent populations (30, 31). This strong link underscores that these are not isolated issues but rather intertwined conditions that likely share underlying vulnerabilities and reinforce one another. Furthermore, our more granular analysis of the depression network itself identified “Negative Mood” as its most central node. This suggests that negative affect is a core component of the depressive experience for these children, not just a peripheral symptom. This finding has significant clinical implications, suggesting that interventions targeting this core affective component may be more effective at destabilizing the entire depressive network than interventions focused on other, less central symptoms.

Our findings offer clear, actionable implications for mental health interventions in rural China. First, the primacy of peer relationships suggests that support systems should expand beyond traditional family-centric models. School-based interventions that focus on fostering positive peer interactions, improving social skills, and establishing peer-support programs may be particularly effective and resource-efficient (32). Second, the centrality of Negative Mood underscores the need for interventions that directly target core depressive symptoms and emotional well-being, rather than focusing solely on behavioral issues (33). Psychoeducational programs that teach children to identify and manage negative affect could provide a critical foundation for improved mental health (34).

While this study provides valuable insights, its findings should be interpreted in light of several limitations, which also point to important directions for future research. First, the cross-sectional design precludes inferences of causality (35); longitudinal studies are needed to untangle the directional relationships between peer quality and mental health. Second, our sample was restricted to fifth- and sixth-grade students from specific provinces. While diverse, these regions predominantly represent rural areas with specific developmental profiles (mostly medium to low development, with the exception of Guangdong), and the findings may not generalize to other age groups, highly urbanized regions, or areas with different socioeconomic characteristics (36). Third, measurement limitations include the use of a single-item measure for parental relationships and the lack of data on the duration of the left-behind experience or the specific role of maternal involvement. Furthermore, as with all self-report measures, the data may be subject to social desirability bias or recall bias, particularly regarding sensitive topics like family dynamics and mental health symptoms. Future research should utilize more comprehensive measures and collect primary data on the temporal dimensions of parental absence. Finally, while network analysis is robust for exploring systemic relationships, future studies could use Structural Equation Modeling (SEM) to formally test the mediation hypotheses proposed here (e.g., peer relationships mediating the effect of left-behind status).

## 5 Conclusion

This study used network analysis to reveal that the mental health of rural Chinese early adolescents is a complex system wherein peer relationships are a more central role than family factors. Depression and anxiety were strongly comorbid, and “Negative Mood” was identified as a core component of the depression network. The impact of left-behind status appears to be indirect, likely buffered by the strength of these proximal peer dynamics. These findings highlight a critical need for interventions in rural communities to shift toward strengthening positive peer interactions and directly addressing core affective symptoms.

## Data Availability

Publicly available datasets were analyzed in this study. This data can be found here: http://doi.org/10.57760/sciencedb.j00001.00464.
